# Glances at the Hospitals

**Published:** 1900-12-29

**Authors:** 


### GLANCES AT THE HOSPITALS.

### THE LIVERPOOL HOSPITAL FOR WOMEN.

THE receipts amounted to £3,043, and the expenditure to £3,485, showing a balance of expenditure over income of £442. The committee remark that this deficiency is all the more serious in view of the increased obligations which they are undertaking; and, further, that unless more liberal support is accorded them, the work of this charity must be, for the future, conducted on more restricted lines. Medical and surgical nurses can be supplied from this hospital at a charge of £1 1s. a week for ordinary cases. A detailed list of surgical cases is not printed with this report, and we have to be content with the information supplied on page 9. It would seem that 768 patients were admitted, and that 536 operations, of which 166 were major, were performed during the year. There were 24 deaths. There were 2,697 out-patients. We venture to hope that more satisfactory tables will accompany the next annual report, as these details are even more essential in a special than in a general hospital.

### THE STOCKPORT INFIRMARY.

THE total receipts for the year amounted to £3,969, and the total expenditure to £3,942. The average cost per in-patient was £4 3s. 9d.; the average cost per bed occupied was £58 15s. 6d.; the average cost per head per week was 6s. 0¾d There were 653 in-patients during the year 1899, and of these 533 were cured, 90 relieved, 31 died, and 45 remained under treatment. There were 882 home patients, and of these 297 were cured, 457 relieved, 17 were made in-patients, 16 were made union patients, 78 died, and 125 were under treatment. There were 1,545 out-patients, and of these 994 were cured, 503 relieved, 40 were made homepatients, 6 died, and 172 remained on the books. There were 1,310 accidents, and of these 1,132 were cured, 174 were made in-patients, and 70 remained on the books. The average stay of each patient in the hospital was 26 days. The tables showing the diseases, accidents, and operations ought to be entirely revised, as they are practically useless for comparison in their present state.

### THE GENERAL INFIRMARY, CHESTER.

The total receipts for the year amounted to £6,289, and the total expenditure to £6,366. In the latter are included one sum of £100 for renovating the Lilian Henderson Ward, and another of £112 spent on the new post-mortem room. A special appeal was made for subscriptions early in the year, and resulted in another £53 being obtained; but there is still a sum of £697 due to the treasurer. On January 1, 1899, the hospital contained 129 patients, and 1,244 were admitted during the year, making a total of 1,373. Of these 764 were discharged cured, 391 relieved, for other reasons 39, died 88, and 91 remained under treatment. In addition to the 88 deaths it should be stated that 26 others took place within 48 hours of admission. The mortality was 4·9 per cent., this calculation being based on the number treated to a termination. The out-patients, including casualties, numbered 4,191, and the home patients 2,327. It is worthy of note that the out-patients showed a decrease of 314 when compared with the number for 1898, and home patients a decrease of 221. The medical and surgical tables are good. Under the various headings they show—first, the total numbers afflicted with the disease named; second, the number cured; third, the number relieved; fourth, the number not relieved; fifth, the number died; and sixth, the number under treatment on the date of the report. As an example let us take gastric ulcer. There were 14 cases: 11 were cured, 1 relieved, and 2 were under treatment. The same system is followed with the operation statistics, and a summary is given of the 88 deaths which occurred among the in-patients. We should like to suggest that next year a short table should be inserted showing the anæsthetic used during the operations.

### THE GUEST HOSPITAL, DUDLEY.

The total income amounted to £3,371, showing a slight falling off from the income of 1898, which was £3,517; but this falling off is fully accounted for by the fact that a special effort was made during the year to clear off the extension debt. The workmen's contributions to the annual income amounted to £788, showing a decrease of £13 on the previous year. The committee say—and we agree with them —that this falling off is greatly to be deplored, "as it was hoped and expected that the income from this source would increase year by year until it reached a sum that would equal the proportion given by working men in other localities." The expenditure for the year was £3,583, which is lower than it was in 1898; nevertheless there was an adverse balance of £81 at the end of the year. At the beginning of the year the hospital contained 66 patients, and 711 were admitted during the year, making a total of 777. Of these 575 were discharged cured, 33 relieved, 35 for other reasons, 60 died, and 74 remained under treatment. These numbers show that 80·87 per cent. of the admissions recovered and that 8·43 per cent. of the admissions died. The number of "emergencies" treated was 318. The medical tables name the diseases and give the number of patients treated in every class, but, unfortunately, do not show the results. It is therefore necessary to refer to Table VI. to learn whether the cases ended in death or not. On the other hand, the table showing the operations of the year is complete. It shows the disease, the date of operation, the nature of the operation, the result thereof, and the surgeon's remarks. We should like to see in the next report a short table naming the anæsthetic used. Several hospitals already give this information.

